# P-1043. Risk Factors for Breakthrough Invasive Mold Infections in Patients with Hematologic Malignancies

**DOI:** 10.1093/ofid/ofae631.1233

**Published:** 2025-01-29

**Authors:** Vaisak O Nair, Aanchal Gupta, Nischal Ranganath, Maria A Mendoza, Ella Nadarevic, Nancy Wengenack, Paschalis Vergidis

**Affiliations:** Mayo Clinic, Rochester, Minnesota; UMass Memorial Medical Center, Worcester, Massachusetts; Mayo Clinic, Rochester, Minnesota; Mayo Clinic, Rochester, Minnesota; Mayo Clinic, Rochester, Minnesota; Mayo Clinic, Rochester, Minnesota; Mayo Clinic, Rochester, Minnesota

## Abstract

**Background:**

Invasive mold infections (IMI) lead to significant morbidity and mortality in patients with hematologic malignancies. We provide patients deemed at higher risk mold active prophylaxis but still observe breakthrough IMIs. The purpose of this study was to evaluate risk factors associated with these breakthrough infections.

**Figure:**
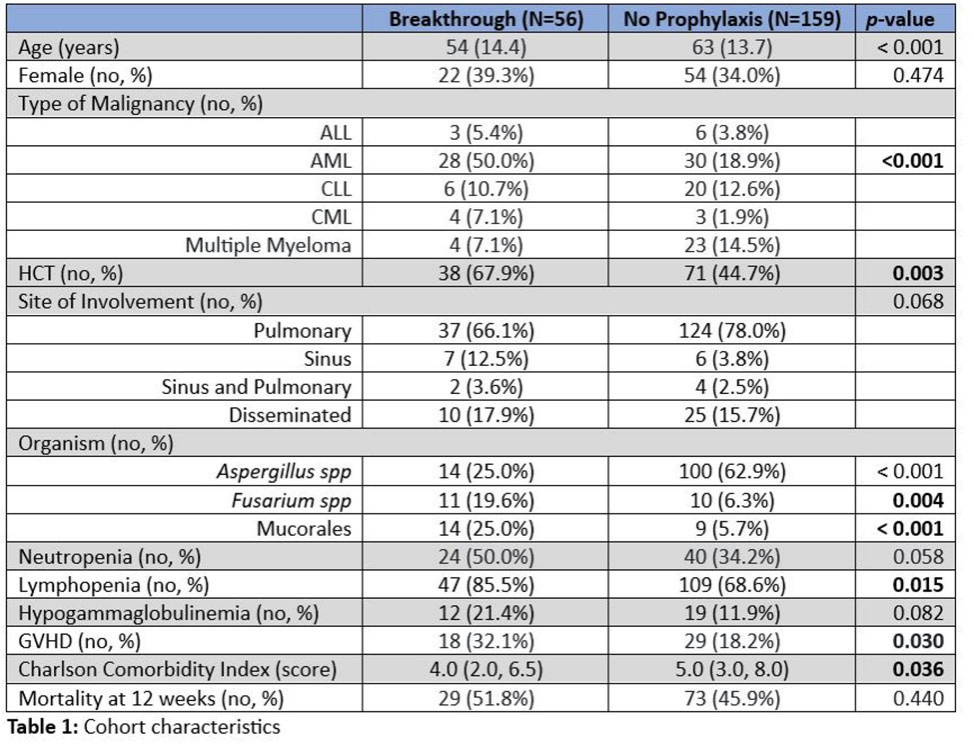

**Methods:**

We conducted a retrospective cohort study of adult hematologic patients at 3 tertiary care medical centers from November 19, 2011 - August 6, 2021. Only IMI cases defined as proven or probable per EORTC/MSG criteria were included. Patients with IMIs who received a minimum of 7 days of mold active prophylaxis were classified as breakthrough infection. We performed logistical regression analysis to identify risk factors associated with breakthrough IMI.

**Figure:**
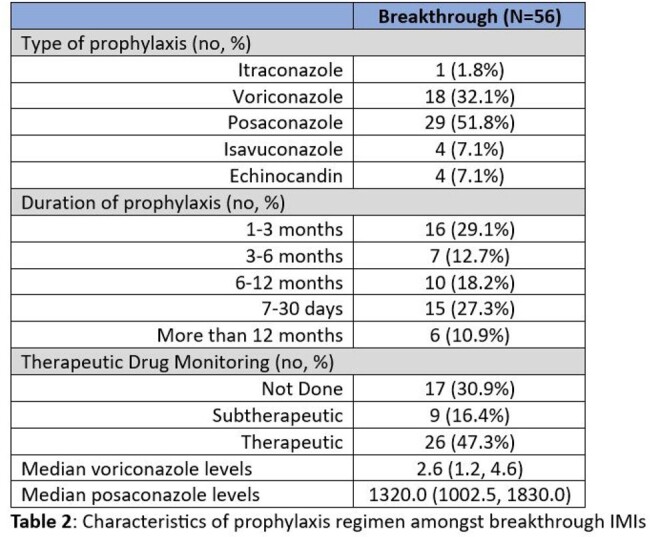

**Results:**

Majority of cases were probable IMIs, with 16 (28.6%) and 25 (15.7%) proven cases amongst breakthrough and non-breakthrough IMIs, respectively. Many variables indicative of weakened host immune system, including neutropenia and hypogammaglobulinemia, were associated with breakthrough IMIs, albeit not statistically significant (Table 1). Multivariate analysis revealed history of AML (OR 4.20, 95% CI 2.03-8.88; *p* < 0.001) and receipt of HCT (OR 3.37, 95% CI 1.46-8.11; *p* = 0.003) to be independent host risk factors for breakthrough IMI. Of all the pathogens, *Fusarium* (OR 4.38, 95% CI 1.56-12.56; *p* = 0.005) and Mucorales (OR 5.59, 95% CI 2.11-15.61; *p* < 0.001) were significantly likely to lead to breakthrough IMIs. 26 (47.3%) of the breakthrough IMIs were on appropriately dosed prophylaxis at the time of infection, with only 9 (16.4%) found to be subtherapeutic (Table 2). Overall, mortality was slightly higher amongst the breakthrough IMIs (51.8% vs 45.9%) but not significantly different (Figure 1).

**Figure:**
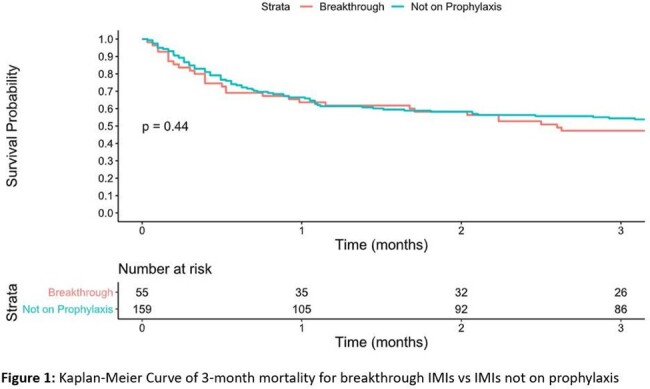

**Conclusion:**

In our cohort of probable/proven IMIs, we did not observe any significant difference in mortality between patients with breakthrough IMIs and those not on mold active prophylaxis. Even with appropriate prophylactic dosing, breakthrough infections occurred, irrespective of agent. Our study demonstrates that host risk factors and the causative pathogen are clinically more pertinent than the prophylaxis regimen in breakthrough IMIs.

**Disclosures:**

**Paschalis Vergidis, MD, MSc**, AbbVie: Advisor/Consultant|Ansun Biopharma: Grant/Research Support|Cidara Therapeutics: Grant/Research Support|F2G: Grant/Research Support|Scynexis: Advisor/Consultant|Scynexis: Grant/Research Support

